# Supplementary material for: Content-rich biological network constructed by mining PubMed abstracts
Source: BMC Bioinformatics. 2004 Oct 8;5:147. doi: 10.1186/1471-2105-5-147 (PMC528731; doi:10.1186/1471-2105-5-147)
Supplement: Additional File 5 — The original Chilibot query results of the term "long-term potentiation (LTP)" and 22 other terms, limiting the latest references analyzed to the years 1990, 1995, 2000, and 2004. [file 1471-2105-5-147-S5.bz2 › chilibotAdditionalFile5/ltp2000/html/AMPA_ARC.html]

 


 **AMPA** and **ARC** 
  
Found 4 abstracts in PubMed,  **4 abstracts were retrieved and analyzed**.  


---

 Search Google  |
 PDF files only 
|  EDU domain only 

---

**Interactive relationship** (e.g. stimulation, inhibition, etc)

**Parallel relationship** (e.g. studied together, co-existance, homology, etc.)

- In contrast,  **AMPA**  receptor GluR1 immunoreactive levels in the magnocellular preoptic area mPOA, the arcuate nucleus  **ARC** , and the suprachiasmatic nucleus SCN were found to be markedly elevated during the time of the LH surge in estradiol progesterone treated castrate rats compared to those of the vehicle only treated castrate rat.  Ref: 7958168 Front Neuroendocrinol, 1994
